# Development and performance evaluation of a recombinant antigen - based ELISA and immunochromatography test for HCMV IgG detection

**DOI:** 10.1186/s12879-026-13424-1

**Published:** 2026-05-16

**Authors:** Mengjiao Lin, Ni Yao, Miaomiao Li, Yushan Xu, Dawei Cui, Jue Xie

**Affiliations:** Department of Blood Transfusion, The First Affiliated Hospital, Zhejiang University School of Medicine, Hangzhou, China

**Keywords:** Cytomegalovirus, Diagnostic performance, Enzyme-linked immunosorbent assay, Colloidal gold immunochromatography assay

## Abstract

**Background:**

Human cytomegalovirus (HCMV) is a common pathogen that poses significant health risks, particularly to immunocompromised individuals. This study aims to improve diagnostic accuracy by developing a novel enzyme-linked immunosorbent assay (ELISA) and colloidal gold immunochromatography assay (GICA) utilizing recombinant HCMV proteins (fusion of pp150 and pp28), which offer improved sensitivity and specificity.

**Methods:**

A recombinant HCMV antigen was synthesized for the detection of CMV IgG antibodies in 573 serum samples collected from Hangzhou, Zhejiang Province. All samples were analyzed using two in-house developed assays (indirect ELISA and GICA), and 528 of these samples were identified as CMV IgG-positive by a commercial ELISA kit. The diagnostic performance and reliability of the two in-house assays were subsequently compared with those of this commercial ELISA kit.

**Results:**

The assays showed no cross-reactivity with other viruses, with a coefficient of variation of less than 10%, indicating excellent reproducibility. Compared to the commercial ELISA kit, the concordance rates of the recombinant antigen-based ELISA and GICA were 98.9% and 99.7%, respectively.

**Conclusion:**

The ELISA and GICA developed in this study demonstrate high sensitivity and specificity, making them suitable for screening CMV IgG antibodies particularly in immunocompromised individuals. These findings highlight the potential of recombinant antigen-based assays to advance in infectious disease diagnostics.

**Clinical trial number:**

Not applicable.

**Supplementary Information:**

The online version contains supplementary material available at 10.1186/s12879-026-13424-1.

## Introduction

Human cytomegalovirus (HCMV) belongs to the Herpesviridae family and infects only humans. It belongs to the double-stranded DNA virus and can cause severe opportunistic and congenital diseases [1]. The significant feature of HCMV infection in the population is its high infection rate. The antibody positive rate of normal adult HCMV is > 90%, and China is also one of the countries with high infection rates [2]. HCMV IgG testing remains clinically valuable because it helps distinguish past infections from recent or acute infection, assess immune status, and guide clinical diagnosis, treatment and public health decision-making—rather than simply determining whether exposure has occurred. This is particularly critical for identifying primary infections in susceptible populations, including pregnant women, immunocompromised patients and organ transplant recipients. In individuals with normal immune function, HCMV infection is mostly asymptomatic or presents with non-specific virus symptoms, with clinical manifestations predominantly observed in special populations. HCMV transmitted via blood transfusion is a major cause of morbidity and mortality of AIDS patients, low-weight newborns, organ transplant patients, tumor patients receiving radiotherapy and chemotherapy, and other patients with decreased immune function [3, 4]. Screening blood donors for HCMV provides an experimental basis for clinical blood transfusion safety, particularly for recipients with immune dysfunction or defects, pregnant women and newborns [5, 6], and also helps clarify the HCMV infection status within donor populations. Clarifying the individual’s HCMV infection status is beneficial for the clinical determination of individual treatment plans. For example, in AIDS patients and organ transplant recipients, HCMV IgG testing helps determine whether their IgM levels require follow-up for timely anti-HCMV treatment [7, 8]. Although HCMV IgG detection reflects only past exposure to the virus and does not indicate active infection, it remains clinically valuable and continues to be in high demand [9]. Currently, the detection of HCMV infection in immunocompromised individuals has become a research hotspot in the medical field both domestically and internationally.

The HCMV genome is ~ 240 kb with estimates of between 165 and 252 open reading frames [10, 11], and the encoded proteins can be designed as recombinant antigens to detect specific IgG antibodies [12]. Previous research reports have shown that CMV envelope phosphoprotein 150 (pp150), phosphoprotein 28 (pp28), envelope glycoprotein B (gB), and nonstructural protein pp52 can be recognized by most CMV IgG positive sera, so they may replace whole virus antigen to detect CMV IgG antibodies [13, 14]. Currently, most commercially available reagents for detecting HCMV IgG antibodies use HCMV whole virus lysates as coating antigens. Their antigen components are complex and may be contaminated by host cell proteins. It is also possible to generate false positive signals by combining them with other viral antibodies of the Herpesviridae family. Although several commercial HCMV IgG detection kits employ recombinant peptides as coating antigens, these assays often exhibit limited stability and suboptimal sensitivity and specificity. However, imported reagents of this type are too expensive. Therefore, we synthesized the recombinant human cytomegalovirus protein (fusion between pp150 + pp28) as a coating antigen to evaluate the sensitivity and specificity of the HCMV IgG detection kit.

The use of enzyme-linked immunosorbent assay (ELISA) and colloidal gold immunochromatography assay (GICA) to screen CMV IgG antibodies in special populations is of great significance for the prevention, management and control of HCMV related diseases and the formulation of relevant policies. ELISA can effectively detect HCMV specific antibodies, this method is easy to operate, fast, highly sensitive, and specific. GICA is a new type of diagnostic technology that uses colloidal gold as a tracer and is combined with an antigen-antibody immune response [15]. It is simple, fast, easy to interpret, does not require any equipment, and has a low cost. We have developed two new detection kits for recombinant antigens using different methods and compared their sensitivity and specificity. This would provide technical support for controlling the related diseases of HCMV.

In this study, we developed an ELISA and an GICA test for detecting HCMV IgG and compared their performance with commercial kits.

## Materials and methods

### Clinical samples

A total of 573 serum specimens were collected from patients in the First Affiliated Hospital, Zhejiang University School of Medicine from 2021 to 2022. After centrifugation, 1 mL of serum was collected and stored at -80 °C. The study was approved by “Clinical Research Ethics Committee of the First Affiliated Hospital, Zhejiang University School of Medicine” in the year 2020 (Clinical Trial Number: 2020 − 856).

### Reagents, equipment, and kits

20 nm gold nanoparticles was purchased from BBI Solutions(Cardiff, Wales, UK); sample pads, absorbent pads, conjugate pads, nitrocellulose membranes (NC), and PVC sheets were purchased from Shanjing (Shanghai, China); potassium carbonate was purchased from Sigma-Aldrich CS (Shanghai, China); triple distilled water was used for preparing colloidal gold experiments. The ultrapure water system was purchased from Millipore Sigma (Burlington, MA, USA); an electronic balance model JY2001 was purchased from Fuyu (Shanghai, China); the AD3200 gold standard diagnostic spotting system were obtained from Biodot (Shanghai, China); CMV IgG ELISA kit was purchased from DIA.PRO Diagnostic Bioprobes Srl (Milan, Italy); ELISA coating solution and stop solutionwas purchased from Solarbio (Beijing, China); ELISA washing solution and TMB developing solution including solution A and solution Bwas purchased from Yuanye (Shanghai, China); 96 hole coating plate was purchased from Nest (Beijing, China). Herpes Simplex Virus IgG Antibody Detection Kit, Epstein-Barr Virus Viral Capsid Antig en (VCA) IgG Antibody Detection Kit, and Hepatitis E Virus IgG Antibody Detection Kit purchased from Beier Biosciences (Beijing, China).

### Production and identification of pp150 + pp28 recombinant protein

According to the published HCMV gene sequence (NC_006273.2) in National Center for Biotechnology Information, the coding sequence of HCMV (pp150 + pp28) gene was determined. The 856–1048 sequence fragment of the pp150 gene and the full-length sequence of the pp28 gene were synthesized and subjected to codon optimization to enhance their expression efficiency. The two fragments were linked by GGGGSGGGG linker, cloned into pet28a vector, and transformed into *E. coli*. After induction with 0.5 mM Isopropyl β-D-Thiogalactoside (IPTG), the bacterial protein was verified by the SDS-PAGE gel (13%), and Coomassie Brilliant Blue staining. Following induction condition optimization, the clone with optimal expression was selected for scale-up culture. It was inoculated into 2 L of LB medium supplemented with 30 µg/mL Kan and cultured at 37 °C with shaking until the OD₆₀₀ reached 0.6 ~ 0.8. IPTG was then added to a final concentration of 0.2 mM, and the culture was induced at 37 °C for 4 h before bacteria were harvested by centrifugation. The collected bacterial pellets were dissolved in 20 mL of lysis buffer and disrupted by sonication (amplitude set at 40%) by 5 pulses of 20s with intervals of 60s on ice [16]. After centrifugation, the supernatant was collected and subjected to preliminary purification using a Ni²⁺ affinity chromatography column. Purity and identity were verified by SDS-PAGE and Western blot [17].

### Establishment of GICA

#### Determination of optimal labeling PH and amount for pp150 + pp28 recombinant protein

Dispense 1.0 mL colloidal gold into five 1.5 mL EP tubes; add 2–10 µL pH regulator (2 µL increments), vortex ≥ 10 s, then add 10 µg recombinant HCMV antigen, vortex ≥ 10 s, and incubate 10 min. Select the tube with stable wine-red color and minimum pH regulator volume (10 µL) as optimal. Add 200 µL blocking solution, vortex ≥ 10 s, incubate 10 min, centrifuge, discard supernatant, and resuspend the pellet in 50 µL labeling complex solution (vortex ≥ 10 s) to obtain the colloidal gold labeled HCMV antigen.

#### Assembly of the test strip

Sheep anti-mouse IgG antibody (0.5 mg/mL) and mouse anti-human IgG antibody (0.4 mg/mL) were sprayed on control line and test line of the nitrocellulose membrane, respectively, with a spray amount of 1.0 µL/cm. After preparation, it needs to be dried at 37 °C for ≥ 6 h. Absorbent and conjugate pads were blocked, dried, and the conjugate pad was coated with the colloidal gold labeled HCMV antigen and colloidal gold labeled mouse IgG [18].

#### GICA reaction principle

For testing, 50 µL serum was added to the sample pad, with results observed within 15 min at room temperature (positive: both lines red; negative: only control line red; invalid: no control line). By capillary action, serum migrates to the conjugate pad; The colloidal gold labeled antigen binds to HCMV IgG in the serum to form an antigen-antibody complex, which is then captured by the mouse anti-human IgG on the test line (red band). Colloidal gold-labeled mouse IgG binds to control line antibodies, confirming valid chromatography.

### Establishment of enzyme-linked immunosorbent assay

#### The optimum concentration of the coating pp150 + pp28 recombinant protein

Two batches of recombinant antigen (2.5 mg/mL) were diluted to 2, 5, 10, and 20 ng/mL with coating solution (100 µL/well) and incubated at 4 °C for 18–24 h. After PBST washing, blocking with 5% BSA, and washing again, serially diluted positive and negative sera were added, followed by enzyme conjugate, TMB development, and stop solution. The optimal coating concentration was 10 ng/mL (strong positive OD: 0.8–1.0; negative OD < 0.1) [19]. Assays were performed per standard ELISA protocols, with results judged by a cut-off value (2.1×average negative serum OD).

#### The optimum concentration of enzyme conjugates

For enzyme conjugate optimization, the antigen was coated at 10 ng/mL, and positive, negative, and blank controls were added. Different dilutions of HRP-labeled anti-human IgG (Solarbio, Beijing) were tested, with 1:25000 selected as optimal (strong positive OD: 0.8–1.0; negative OD < 0.1).

#### The optimal sample dilution ratio

Dilute the strongly positive serum, weakly positive serum, and negative serum samples with sample diluent starting from the original dilution ratio, with dilutions of 1:5 to 1:80, mix well, cover with sealing adhesive, and incubate at 37 ℃ for 30 min before washing the plate. Add the optimal concentration of enzyme binding compound 100µL/well, cover with sealing adhesive, incubate at 37 ℃ for 30 min, and then wash the board. The TMB chromogenic solution consists of two components, namely solution A and solution B. Add solution A and solution B respectively 50µL/well; after 15 min of dark coloration at 37℃, add the termination solution of 50µL/well and read the results of the enzyme-linked immunosorbent assay. 1:10 as the optimal sample dilution ratio was chosen (same OD criteria).

#### ELISA reaction principle

Verify the validity of the experiment through absorbance measurement results. If the following conditions are met: (i) blank control A value ≤ 0.08, (ii) 0.8 < quality control serum A value < 2.4, (iii) negative control A value < 0.12, the experiment is valid; The detection result is determined by the critical value, which is 2.1 times the average A value of negative serum. When the A value of the test sample is less than the critical value, it is judged as negative, and when the A value of the sample is greater than or equal to the critical value, it is judged as positive.

### Evaluation of the GICA and ELISA performance

To overcome the limitation of the absence of an ideal gold standard, a latent class model was applied to analyze the combined results from our self-developed ELISA kit, the GICA test strip, and a commercial diagnostic kit. This model postulates a latent, unobservable true disease status, which is inferred by synthesizing the results of the three detection assays, while the sensitivity and specificity of each assay are estimated concurrently. All model computations and analyses were implemented in Mplus software (version 8.3). Confidence intervals (CI) of 95% were applied to the data on sensitivity, specificity, and cross-reactivity. SPSS 26.0 software was used to perform the analysis. *p*-values < 0.05 was considered statistically significant.

### Clinical sample application

The GICA for detecting CMV IgG antibodies prepared in this study, and the ELISA kit were used to test 300 serum samples included 283 positive samples and 17 negative samples for CMV IgG antibodies and 273 serum samples included 245 positive samples and 28 negative samples, respectively.

### Latent Class Analysis (LCA)

LCA was employed to evaluate the diagnostic performance of the in-house ELISA, in-house GICA, and commercial DIA.PRO ELISA kit, given the absence of a gold standard for HCMV IgG detection. Detailed model specifications, prior settings, key assumptions, and uncertainty assessments were supplemented to ensure reproducibility, as described below:

Prior distributions were specified to reflect the a priori knowledge of assay performance while minimizing bias. For the commercial DIA.PRO ELISA kit, informative beta priors were assigned to sensitivity and specificity based on manufacturer’s technical specifications and published literature [20]: sensitivity was modeled with a beta distribution (α = 10, β = 1), reflecting high confidence in the commercial assay’s diagnostic accuracy, and specificity was also assigned a beta distribution (α = 10, β = 1). In contrast, non-informative beta priors (α = 1, β = 1; i.e., uniform distribution) were used for the in-house ELISA and GICA to avoid introducing prior bias, as no a priori performance data were available for these newly developed assays.

Three key assumptions underpinning the LCA model were explicitly stated and validated: [1] The latent true disease status (HCMV IgG-positive or -negative) was assumed to be independent of the detection methods, ensuring that assay performance estimates were not confounded by method-related biases; [2] Conditional independence of the three assays was assumed, meaning that the error of one assay (false positive or false negative) was not correlated with the errors of the other two assays, given the latent disease status; [3] The study population (HCMV-infected and non-infected individuals recruited from the First Affiliated Hospital of Zhejiang University School of Medicine) was assumed to be representative of the target population, ensuring the generalizability of the model results.

The LCA model was specified with two latent classes, corresponding to HCMV IgG-positive and HCMV IgG-negative statuses, respectively. The primary parameters to be estimated included the sensitivity and specificity of each of the three assays. Maximum likelihood estimation was performed using Mplus software (version 8.3), with model convergence determined by two criteria: log-likelihood convergence and parameter standard errors < 0.05.

Uncertainty in the LCA results was assessed by calculating 95% confidence intervals (CIs) for the estimated sensitivity and specificity of each assay, providing a robust measure of the precision of the diagnostic performance estimates.

## Results

### Design of quimeric gene pp150 + pp28

**Fig. 1:**
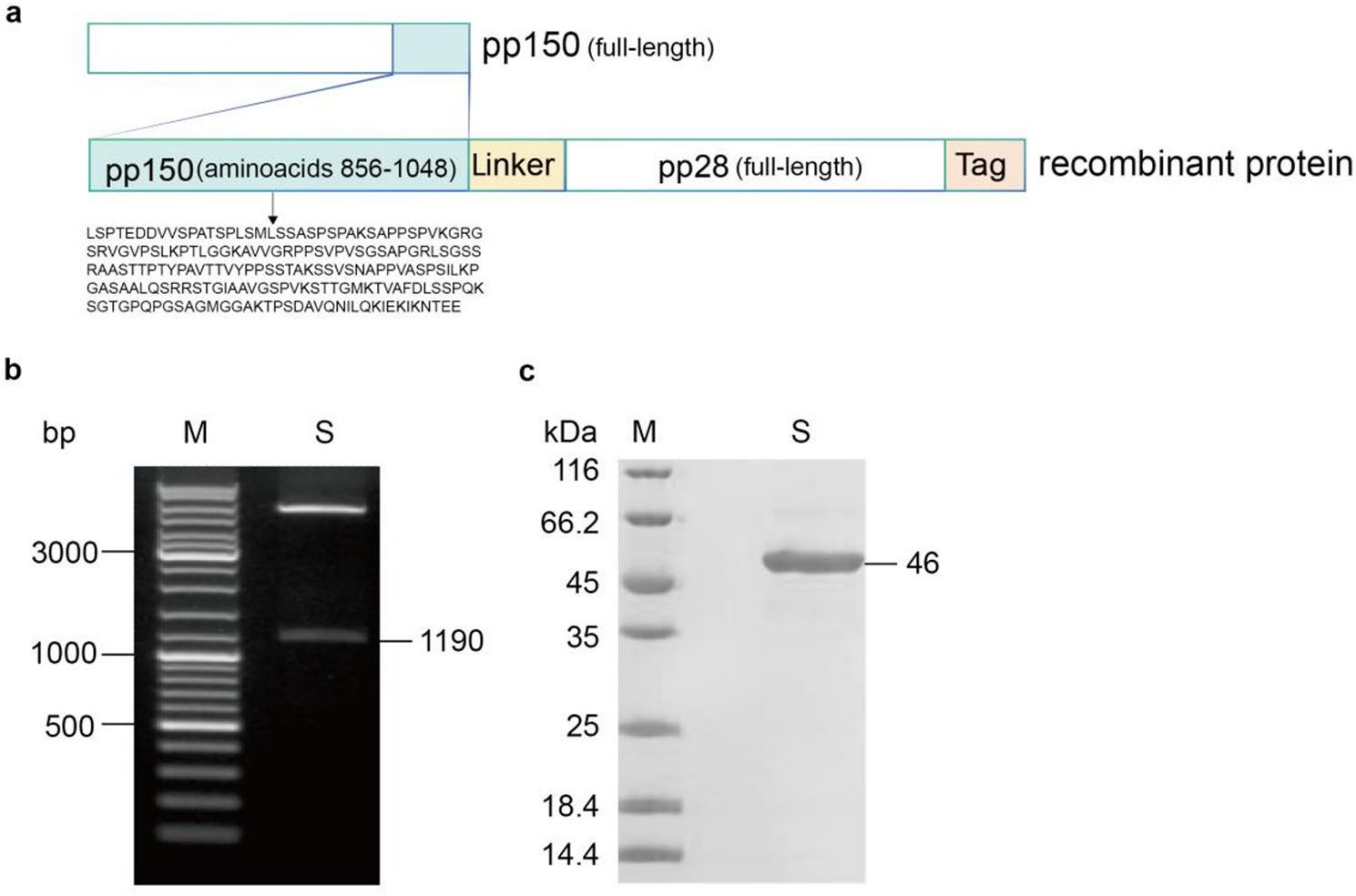
Identification and production of pp150 + pp28 recombinant protein. (**a**) Schematic diagram of pp150 + pp28 recombinant protein, showing the domain organization of the chimeric construct. Linker, GGGGSGGGG; Tag, His-tag. **(b)** Agarose gel analysis of pp150 + pp28 recombinant protein. **(c)** SDS-PAGE analysis of pp150 + pp28 recombinant protein. M, Marker; S, Sample

We first designed the gene fragment encoding the pp150 (aminoacids 856–1048) and full-length pp28, which were linked as a fusion gene(Fig. 1a). The PCR product, synthesized and provided by Sangon Biotech, yielded a specific band at approximately 1,190 bp as visualized by agarose gel electrophoresis(Fig. 1b).

### Production of pp150 + pp28 recombinant protein

Following IPTG induction, bacterial cultures harboring either the empty vector pET-28a or the recombinant plasmid pET28a+pp150 + pp28 were analyzed by SDS-PAGE. No protein expression was detected in the pET28a control, whereas the pET28a+pp150 + pp28 transformants exhibited a distinct protein band at ~ 46 kDa upon induction. The observed molecular weight matched the predicted size of the pp150 + pp28 fusion protein, which accounted for approximately 40% of the total bacterial protein content. The purified pp150 + pp28 recombinant protein had relative molecular weights of approximately 46 kDa, respectively (Fig. 1c), indicating that the purified antibody band was correct and the purity was high.

### Cross‑reactivity assessment of the pp150 + pp28 recombinant antigen in ELISA assay

The established indirect ELISA method was employed to detect three IgG-positive serum samples each for Herpes simplex virus (HSV), Hepatitis E virus (HEV), Epstein-Barr virus (EBV) and HEV + EBV, and evaluate potential cross-reactivity with other viral antibodies. The serum for cross-reactivity analysis were pre-validated as HSV, HEV or EBV IgG-positive using commercially available, certified diagnostic ELISA kits (Herpes Simplex Virus IgG Antibody Detection Kit, Epstein-Barr Virus Viral Capsid Antig en (VCA) IgG Antibody Detection Kit, and Hepatitis E Virus IgG Antibody Detection Kit), thus ensuring the reliability of specificity assay results. The absorbance values (A_450_) were 0.097, 0.084, an 0.101, respectively, all below the cutoff value of 0.135. These results demonstrate no detectable cross-reactivity with HSV-, HEV-, EBV-, or HEV + EBV-positive sera, confirming the high specificity of this assay.

The developed indirect ELISA was used to detect five known HCMV IgG-positive serum samples. The results demonstrated excellent reproducibility, with intra-assay coefficients of variation (CVs) ranging from 2.3% to 6.5% and inter-assay CVs between 1.6% and 6.9%. All CV values were below the 10% threshold, indicating robust assay performance (Table 1).

**Table 1:** Intra group and inter group coefficients of variation of ELISA method

Sample number	Intra-group comparison		Inter-group comparison	
	x ± s	CV(%)	x ± s	CV(%)
1	1.460 ± 0.046	3.2	1.457 ± 0.024	1.6
2	1.080 ± 0.053	4.9	1.067 ± 0.024	2.2
3	0.700 ± 0.037	5.3	0.688 ± 0.048	6.9
4	0.571 ± 0.037	6.5	0.581 ± 0.039	6.7
5	0.307 ± 0.007	2.3	0.289 ± 0.015	5.4

CV, coefficient of variation; x, mean optical density value; s, standard deviation

### The structure and results of the GICA

**Fig. 2:**
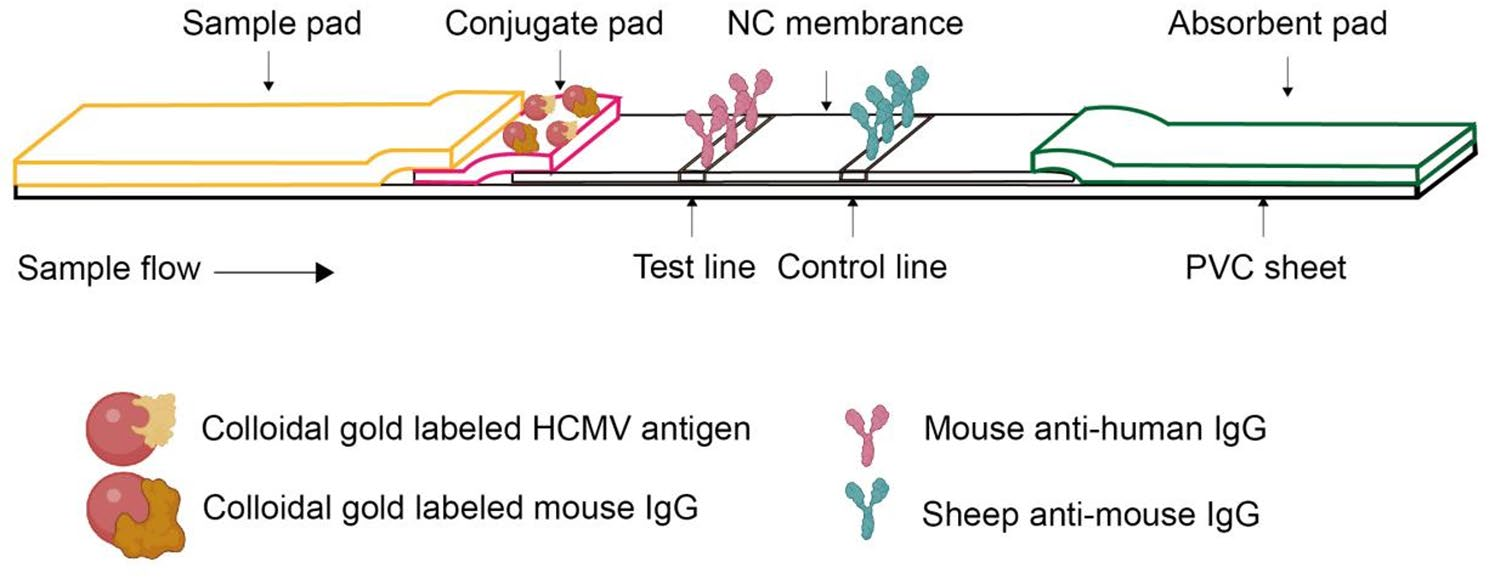
Schematic illustration of GICA. The serum is loaded onto the sample pad and the colloidal gold labeled HCMV antigen is added onto the conjugate pad. Mouse anti-human IgG is immobilized as the test line in the NC membrane. Sheep anti-mouse IgG is used as the control line. Following the application of a serum sample containing specific HCMV IgG and non-specific IgG onto the NC membrane, the labeled HCMV IgG complex is captured by the Mouse anti-human IgG on the test line, resulting in a red band. The lableded mouse IgG complex are captured by the sheep anti-mouse on the control line, resulting in a red band

The GICA was mainly constructed from a sample pad, absorbent pad, conjugate pad, NC membrane, and a PVC sheet (Fig. 2).

**Fig. 3:**
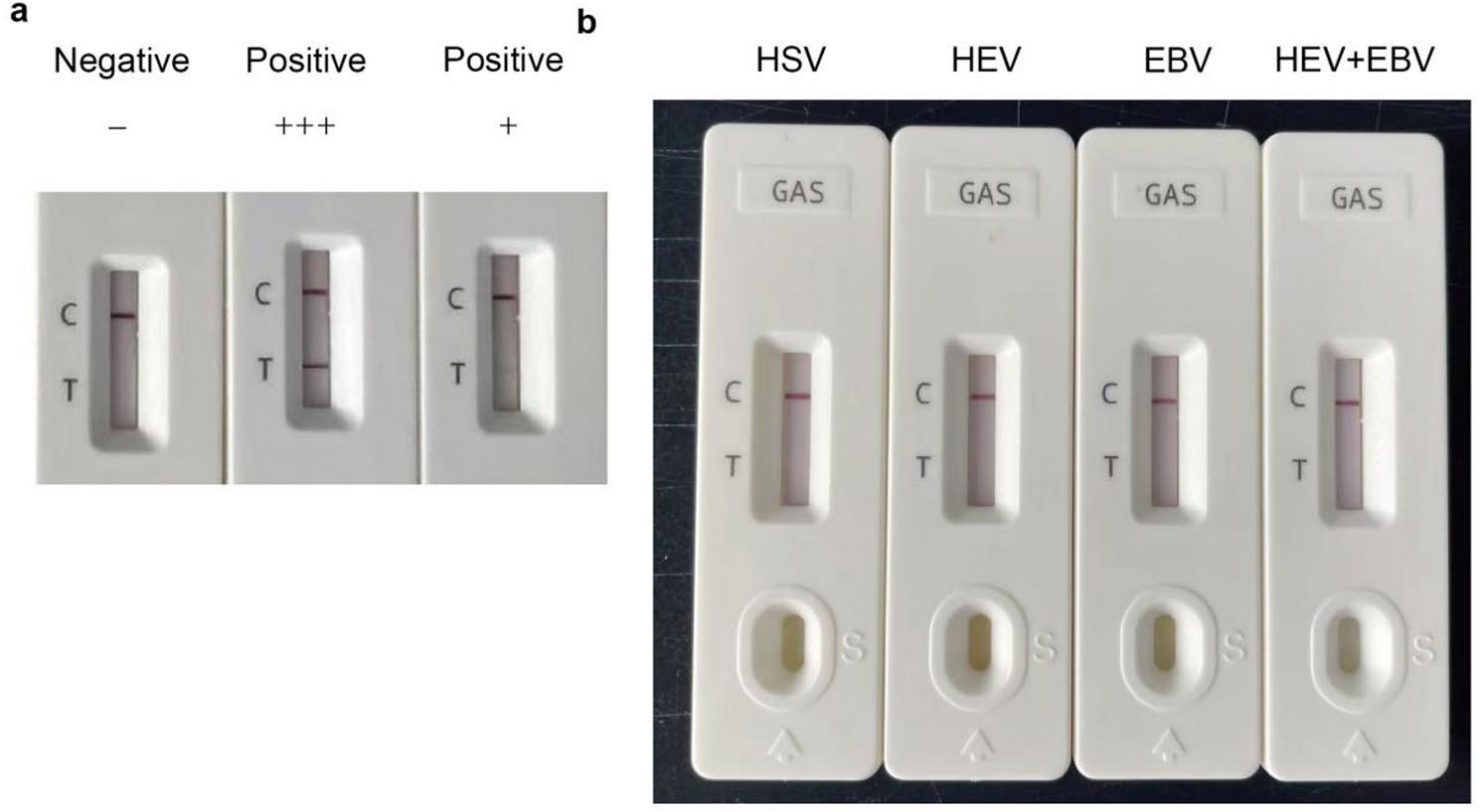
A typical diagram of CMV IgG detected by GICA and specificity analysis. **(a)** Patients serum sample was used as the negative test results and positive test results included + and ++. **(b)** Using GICA strips to detect HSV-, HEV-, EBV-, and HEV + EBV-positive serum samples to determine the assay’s specificity. C, control line. T, test line

The recombinant antigens, synthesized from the pp150 gene (aminoacids 856–1048) and the full-length pp28 sequence, were coated onto the colloidal gold test strips. When testing clinically negative serum samples, only the control line (C-line) developed color, whereas both the control and test lines (T-line) were visible for positive clinical sera. These results demonstrate the feasibility of the proposed method (Fig. 3a**)**. The pre-established colloidal gold test strips were employed to detect three IgG-positive serum samples each for HSV, HEV, EBV and HEV + EBV, and evaluate potential cross-reactivity with other viral antibodies. Results demonstrated coloration exclusively at the control line, confirming no cross-reactivity with HSV, HEV, EBV, or HEV + EBV positive sera. These findings validate the method’s high specificity (Fig. 3b).

### Performance of the ELISA and GICA with protein pp150 + pp28

The performance of the in-house ELISA were compared with that of the commercial DIA.PRO ELISA kit. Both kits were used to analyze serum samples, with results classified as either negative or positive. The results demonstrated no statistically significant difference between the two assay kits (McNemar’s test, *p*-value = 0.625). Compared to the commercial kit, our ELISA assay exhibited: Sensitivity: 99.6% (95% *CI*: 97.4%-99.9%), Specificity: 92.9% (95% *CI*: 75.0%-98.8%), Positive predictive value: 99.2% (95% *CI*: 96.8%-99.9%), Negative predictive value: 96.3% (95% *CI*: 79.1%-99.8%). The overall concordance rate between the two methods was 98.9%, indicating excellent diagnostic agreement **(**Table 2**)**.

The performance of the in-house GICA assay were compared with that of the commercial DIA.PRO ELISA kit. Both kits were used to analyze serum samples, with results classified as either negative or positive. The results demonstrated no statistically significant difference between the two assay kits (McNemar’s test, p-value = 0.317). Compared to the commercial kit, our GICA assay exhibited: Sensitivity: 100% (95% CI: 98.3%-100%), Specificity: 94.1% (95% CI: 69.2%-99.7%), Positive predictive value: 99.6% (95% CI: 97.7%-99.9%), Negative predictive value: 100% (95% CI: 75.9%-100%). The overall concordance rate between the two methods was 99.7%, indicating excellent diagnostic agreement (Table 3).

**Table 2:** Comparison between recombinant antigen-based ELISA kit and commercial diagnostic kit

		Italy DIA.PRO ELISA kit		
		No. of positive	No. of negative	No. of total
Recombinant fusion Ag-ELISA kit	No. of positive	244	2	246
	No. of negative	1	26	27
	No. of total	245	28	273

**Table 3:** Comparison between recombinant antigen-based GICA strips and commercial diagnostic kit

		Italy DIA.PRO kit		
		No. of positive	No. of negative	No. of total
Recombinant fusion Ag-GICA strips	No. of positive	283	1	284
	No. of negative	0	16	16
	No. of total	283	17	300

When the recombinant fusion Ag-developed ELISA kit and GICA test strips were respectively compared with the Italy DIA.PRO kits, the number of inconsistent cases was relatively small (3 cases, 1 case). McNemar’s test showed *p* > 0.05 for both comparisons, indicating no statistically significant difference. These findings suggest that the discrepancies between the in-house developed reagents and the reference reagents are attributed solely to random errors, with no essential methodological differences.

### Accuracy of the three diagnostic tests

The accuracy of Recombinant fusion Ag-ELISA kit, GICA strips, and Italy DIA.PRO ELISA kit were estimated by latent LCA using informative priors. The commercial Italy DIA.PRO ELISA kit exhibited the highest sensitivity (97.9%), which was superior to that of the recombinant fusion Ag-developed ELISA (94.2%) and GICA strips (96.1%). Although the sensitivity of GICA strips was slightly higher than that of the recombinant fusion Ag-ELISA kit, their 95% CIs might overlap, indicating no statistically significant difference in sensitivity between the two assays. All three assays showed good specificity (> 93%). The specificity of recombinant fusion Ag-GICA strips (95.2%) was slightly higher than that of the ELISA kit (92.9%). the two in-house assays showed high specificity (> 92.9%), with a low risk of false positive results (Table 4**)**.

**Table 4:** Posterior median and 95% *CI* of sensitivity and specificity for the recombinant fusion Ag-ELISA kit, the recombinant fusion Ag-GICA strips and Italy DIA.PRO ELISA kit obtained from LCA

	Informative priors	
	Median	95% CI
**Sensitivity**		
Recombinant fusion Ag-ELISA kit	0.942	0.921–0.964
Recombinant fusion Ag-GICA strips	0.961	0.942–0.973
Italy DIA.PRO ELISA kit	0.979	0.971–0.998
**Specificity**		
Recombinant fusion Ag-ELISA kit	0.929	0.886–0.967
Recombinant fusion Ag-GICA strips	0.952	0.914–0.972
Italy DIA.PRO ELISA kit	0.976	0.957–0.991

## Discussion

Many recombinant antigens have been produced and characterized for their ability to bind CMV specific antibodies [21, 22]. However, the exact composition of the antigenic mixture representative of the entire complex of CMV antigens to be used for detecting CMV antibodies remains under investigation. HCMV, a herpesvirus with humans as its sole natural host, exhibits widespread seroprevalence globally. It typically establishes lifelong latency without overt clinical manifestations. However, under immunocompromised conditions, viral reactivation can lead to severe or fatal outcomes [23]. Timely diagnosis and effective intervention are critical to mitigate disease progression. Current laboratory diagnostics primarily rely on ELISA and PCR [24]. While PCR is sensitive, it is limited by technical complexity, instrumentation requirements, and high costs, which hinder its use for large-scale screening in resource-limited primary healthcare facilities [25]. This study focuses on establishing and comparing ELISA and colloidal gold-based immunoassays for HCMV IgG detection, with the goal of addressing practical limitations of existing methods.

Currently, a small number of commercial HCMV IgG detection kits still employ whole-virus lysates as coating antigens, which have inherent drawbacks, including complex antigenic components, potential contamination with host cell proteins, and cross-reactivity with antibodies against other herpesviruses, thereby increasing the risk of false-positive results. Alternative kits using recombinant HCMV peptides offer cost-effective production but may have demonstrate suboptimal stability, sensitivity, and specificity. Imported versions of such kits remain prohibitively expensive. To address these limitations, we developed assays using a recombinant pp150 + pp28 fusion protein as the coating antigen, a strategy intended to facilitate standardization through precise antigenic composition and improved batch-to-batch consistency.

Previous studies have demonstrated that the seropositivity rates detected by fused multiepitope antigens are generally higher than those detected by single-peptide antigens. Prior literature reported that the recombinant peptide pp150 showed significantly higher concordance with comparator methods in detecting serum IgG antibodies compared to other recombinant peptides (pp52, pp28, and gB), while pp28 also showed higher concordance than pp52 and gB [26]. Based on these findings, we design an expression plasmid containing gene fragments to produce the fusion protein with pp150 and pp28 from HCMV, as co-expression of these two fragments offers practical advantages: it eliminates the need for separate preparation or synthesis of the two proteins and optimization of their mixing ratios, thereby improving operational convenience and reducing costs; additionally, the fusion of immunodominant epitopes from both proteins may mitigate nonspecific reactions and address instability issues associated with small peptide antigens. To our knowledge, no published studies have reported the fusion expression of pp150 and pp28 for HCMV IgG detection, making this a novel approach to antigen design for HCMV serological assays.

ELISA and colloidal gold immunochromatographic detection technologies are well-established for serological diagnosis, and leveraging the advantages of both methods—along with the characteristics of the pp150 + pp28 fusion antigen—we established ELISA and GICA assays for HCMV IgG detection that can be used for convenient and rapid assessment of HCMV infection status. In this study, a gold standard-free LCA was employed to objectively estimate the sensitivity and specificity of three HCMV IgG detection kits, followed by a direct comparative analysis, without the need for a conventional gold standard. The results showed that the in-house developed GICA test strips and in-house ELISA both exhibited favorable sensitivity and specificity; the in-house GICA test strips achieved a well-balanced performance between analytical efficacy and operational convenience, while the in-house ELISA demonstrated prominent cost-effectiveness. This study thus provides robust statistical evidence for the rational selection of HCMV IgG detection kits suited to different application scenarios. A key finding of this study is that both developed assays showed high agreement with a commercial ELISA kit, with a concordance rate exceeding 98%. This level of concordance indicates that the pp150 + pp28 fusion antigen performs consistently with the antigen used in the commercial kit, supporting its potential utility in HCMV IgG detection. The results also suggest that combining multiple immunodominant antigens may be a useful strategy for optimizing the performance of ELISA or GICA assays, as observed in the high concordance between our fusion antigen-based assays and the commercial comparator.

While ELISA and GICA are widely used for serological screening, both methods have inherent limitations: GICA is rapid but may be subject to subjective interpretation and lower sensitivity/specificity compared to ELISA, while ELISA is more precise but labor-intensive and still vulnerable to cross-reactivity and non-specific binding. Our study also had limitations, including selection bias from a clinical cohort with high HCMV seroprevalence and the absence of a true gold-standard reference method, which prevents definitive differentiation between true infections and false positives. False-positive results, potentially driven by cross-reactivity, or non-specific binding, may lead to misclassification of infection status. Thus, all positive screening results from the developed ELISA and GICA assays should be considered preliminary and require confirmation by more specific methods (e.g., nucleic acid amplification testing, western blotting, or high-specificity ELISA) to ensure accurate diagnosis. We therefore recommend that positive results from either assay be confirmed by an alternative method to ensure diagnostic accuracy. Additionally, continuous monitoring of HCMV seroprevalence is important for understanding the burden of infection in high-risk populations such as pregnant women, organ transplant patients, and severely immunosuppressed individuals.

## Conclusion

This study developed ELISA and GICA assays for HCMV IgG detection using a recombinant pp150 + pp28 fusion protein as the coating antigen, with optimized coating and preparation steps to enhance assay consistency. The developed ELISA and GICA assays showed high concordance with a commercial ELISA kit from an Italian company, indicating that the pp150 + pp28 fusion antigen-based assays perform consistently with the commercial kit. The fusion of pp150 + pp28 provides a practical approach to antigen design that improves operational convenience and standardization, and the high concordance with the commercial comparator supports the potential utility of this fusion antigen in HCMV serological diagnostics. Limitations of this study, including selection bias, high seroprevalence in the study cohort, and the absence of a true gold-standard reference, should be considered when interpreting these results.

## Supplementary Information

Below is the link to the electronic supplementary material.

Supplementary Material 1

Supplementary Material 2

## Data Availability

All data supporting the findings of this study are available within the paper and its Supplementary Information.

## Abbreviations

HCMV: Human cytomegalovirus
ELISA: Enzyme-linked immunosorbent assay
GICA: Colloidal gold immunochromatography assay
pp150: Phosphoprotein 150
pp28: Phosphoprotein 28
gB: Envelope glycoprotein B
IPTG: Isopropyl β-D-Thiogalactoside
NC: Nitrocellulose membranes
PCR: Polymerase Chain Reaction
HSV: Herpes simplex virus
HEV: Hepatitis E virus
EBV: Epstein-Barr virus
CVs: Coefficients of variation
C-line: Control line
T-line: Test line
