# Supplementary figures and images for: Development and performance evaluation of a recombinant antigen - based ELISA and immunochromatography test for HCMV IgG detection
Source: BMC Infect Dis. 2026 May 16;26:1300. doi: 10.1186/s12879-026-13424-1 (PMC13360036; doi:10.1186/s12879-026-13424-1)

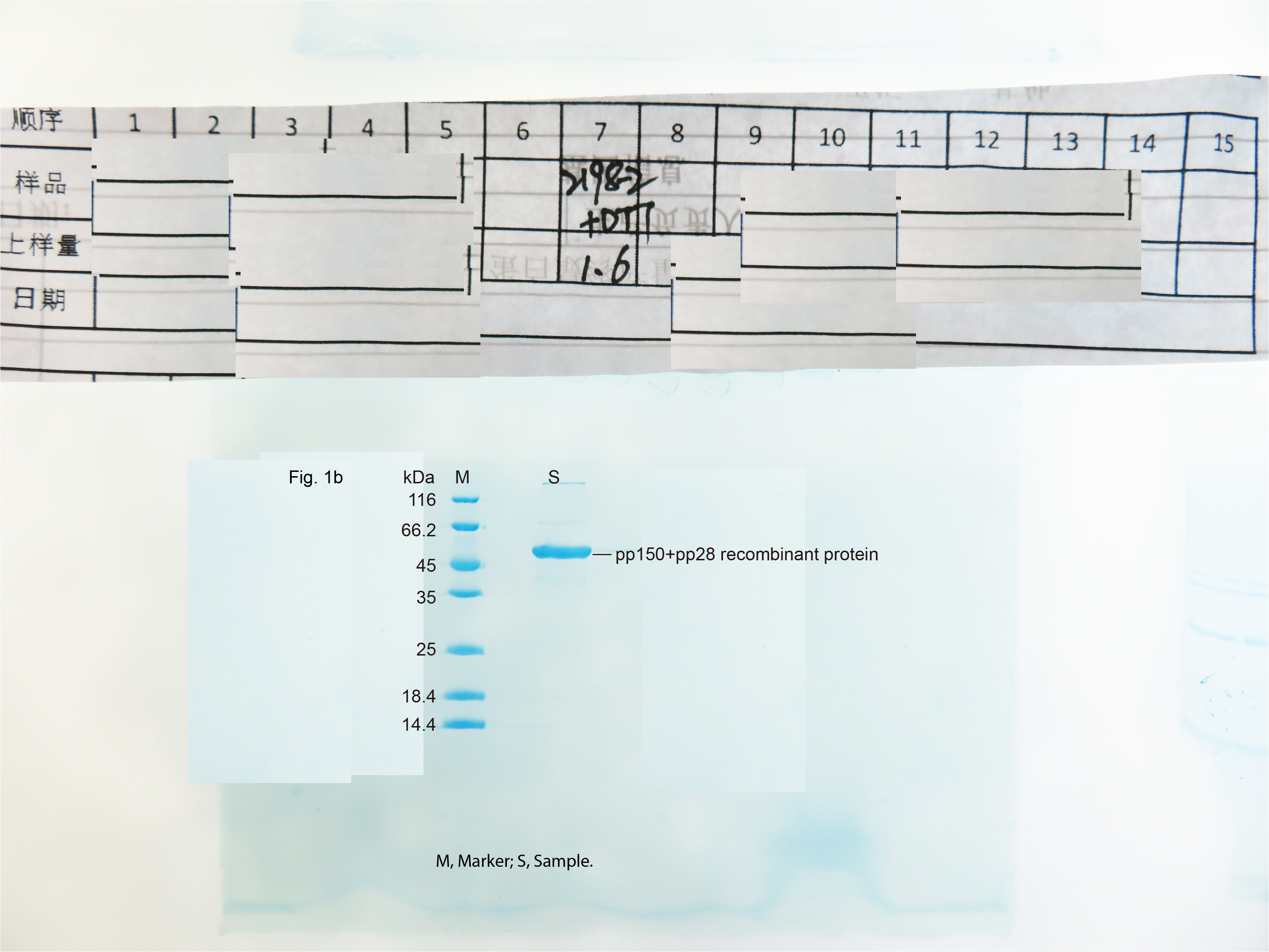

Supplement: Supplementary file 1 — Supplementary Material 1 [file 12879_2026_13424_MOESM1_ESM.jpg]

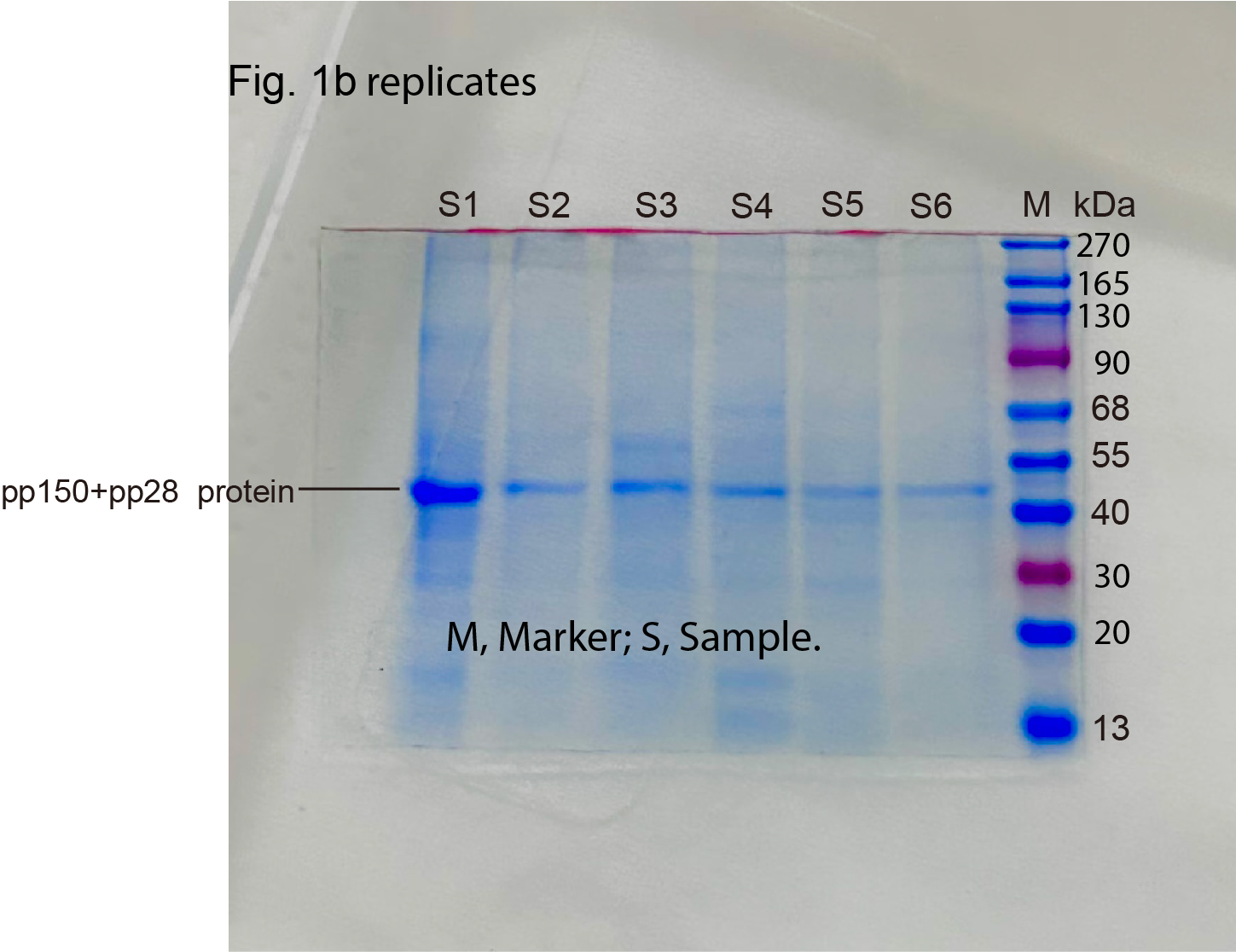

Supplement: Supplementary file 2 — Supplementary Material 2 [file 12879_2026_13424_MOESM2_ESM.jpg]
